# Using OCT Angiography to Predict Diabetic Retinopathy Progression and Vision Decline in a Multiethnic Cohort

**DOI:** 10.1016/j.xops.2026.101111

**Published:** 2026-02-24

**Authors:** Qianhui Yang, Kelvin Y.C. Teo, Yueheng Hong, Bingyao Tan, Rose Tan, Leopold Schmetterer, Chan Choi Mun, Gemmy Chui Ming Cheung, Tien Yin Wong, Gavin Siew Wei Tan

**Affiliations:** 1 Tianjin Key Laboratory of Retinal Functions and Diseases, Tianjin Branch of National Clinical Research Center for Ocular Disease, Eye Institute and School of Optometry, Tianjin Medical University Eye Hospital, Tianjin, China; 2 Singapore National Eye Centre, Singapore Eye Research Institute, Singapore; 3 SNEC Ocular Reading Centre, Singapore National Eye Centre, Singapore; 4 Duke-NUS Medical School, National University of Singapore, Ophthalmology and Visual Sciences Academic Clinical Program, Singapore; 5 SERI-NTU Advanced Ocular Engineering (STANCE) Program, Singapore; 6 Wilmer Eye Institute, Johns Hopkins Medicine, Baltimore, Maryland; 7 School of Chemistry, Chemical Engineering and Biotechnology, Nanyang Technological University, Singapore; 8 Department of Clinical Pharmacology, Medical University of Vienna, Vienna, Austria; 9 Center for Medical Physics and Biomedical Engineering, Medical University of Vienna, Vienna, Austria; 10 Fondation Rothschild, Paris, France; 11 Aier Eye Hospital Group, Changsha, China; 12 Beijing Visual Science and Translational Eye Research Institute (BERI), School of Clinical Medicine, Beijing Tsinghua Changgung Hospital, Tsinghua Medicine, Tsinghua University, Beijing, China; 13 Beijing Key Laboratory of Intelligent Diagnostic Technology and Devices for Major Blinding Eye Diseases, Beijing, China

**Keywords:** Diabetic retinopathy, OCTA, Prediction, Progression, Cohort study

## Abstract

**Purpose:**

To determine whether baseline retinal and choriocapillaris (CC) vascular features measured by OCT angiography (OCTA) can predict diabetic retinopathy (DR) progression or visual acuity (VA) decline in a multiethnic longitudinal cohort.

**Design:**

Prospective longitudinal cohort study.

**Subjects:**

A total of 309 eyes from 192 patients with type 2 diabetes mellitus were recruited from a tertiary eye center in Singapore.

**Methods:**

All participants underwent 3 × 3 mm swept-source OCTA imaging at baseline. Quantitative vascular parameters—including vessel density (VD), perfusion density (PD), and CC flow deficit percentage—were obtained from the superficial and deep retinal layers and from the CC. Larger retinal arterioles and venules were analyzed separately from capillary networks. Diabetic retinopathy progression was defined as a ≥2-step increase on the ETDRS severity scale over 2 years, while VA decline was defined as >1-line reduction in best-corrected VA. Logistic regression and area under the receiver operating characteristic curve (AUC) analyses were used to evaluate predictive performance.

**Main Outcome Measures:**

Progression of DR and VA decline over 2 years, as predicted by baseline OCTA metrics.

**Results:**

Over 2 years, 49 eyes (15.9%) demonstrated DR progression. Significant predictors in the superficial layer included larger foveal avascular zone (FAZ) area (odds ratio [OR] = 6.612; *P* = 0.034), longer perimeter (OR = 1.583; *P* = 0.002), poorer circularity (OR = 3.23; *P* = 0.019), higher large-vessel PD (OR = 1.561; *P* < 0.001) and VD (OR = 2.878; *P* < 0.001), and lower whole-vessel VD (OR = 0.798; *P* = 0.010). Adding FAZ perimeter and large-vessel VD improved prediction accuracy (AUC increased from 0.709–0.822). For VA loss, higher superficial large-vessel PD (OR = 1.609; *P* = 0.002) and lower capillary PD (OR 0.8; *P* = 0.010) were significant predictors, improving AUC from 0.602 to 0.702.

**Conclusions:**

Enlargement and irregularity of the FAZ, along with increased superficial large VD, independently predict DR progression. Incorporating FAZ and large-vessel OCTA parameters enhances prediction models for both DR worsening and vision decline in patients with diabetes.

**Financial Disclosure(s):**

Proprietary or commercial disclosure may be found in the Footnotes and Disclosures at the end of this article.

Diabetes mellitus (DM) is a major global public health burden, with the number of affected adults projected to reach approximately 642 million by 2040.^1^ The most common microvascular complication of the disease, diabetic retinopathy (DR), and diabetic macular edema (DME) are the major causes of blindness among the working-age population.^2^ Progression of DR can lead to serious complications such as vitreous hemorrhage, retinal detachment, and neovascular glaucoma which may lead to visual impairment.^3^

Traditional risk factors for DR progression are primarily systemic, including diabetes duration, glycemic control, hypertension, and microalbuminuria; however, these factors account for only a limited proportion (approximately 11%) of the variability in DR progression.^4^^,^^5^ Moreover, DR progression may be asymmetric between eyes, and systemic risk factors can vary significantly between individuals and over time.^6^ These observations highlight the need for more reliable biomarkers to better predict disease progression.

Annual screening for DR in patients with DM is recommended by international guidelines to facilitate early detection and management of the disease.^7^^,^^8^ Once DR develops, the risk of progression increases, necessitating referral to an ophthalmologist for more frequent monitoring and management.^9^^,^^10^ However, predicting how rapidly DR progresses is currently imprecise and follow-up intervals are generally based on DR severity alone.

Identifying baseline imaging biomarkers capable of predicting future disease progression is key, as they can enable more effective and targeted prevention and management of the disease. As noninvasive tools, OCT and OCT angiography (OCTA) have been utilized in several researches to identify vascular biomarkers which may predict the progression of DR, offering new insights into the potential reclassification of DR and risk stratification.^11^^,^^12^

Imaging biomarkers on fluorescein angiography have been shown to better predict DR progression than severity on standard color photographs; however, the use of intravenous dye and prolonged imaging acquisition times are major barriers to broader adoption.^13^^,^^14^ OCT angiography has been utilized as a noninvasive tool to assess the impact of vascular conditions on specific layers of the retina and choroid.^15^ Current OCTA technology allows the visualization and quantification of capillary perfusion and other microvascular change in both the retina and choroid.^16^ Several studies have demonstrated that OCTA has an exceptional ability to identify early microvascular changes in patients with DR and may have greater sensitivity to diabetic macular ischemia.^17^, ^18^, ^19^ The choroid provides the majority of oxygen to the outer retinal layers, including the photoreceptors and the retinal pigment epithelium, and serves as the primary source of oxygenation for photoreceptors in the foveola. Accordingly, this suggests that choriocapillaris (CC) perfusion has the potential to influence visual acuity (VA) and the progression of DR.^20^^,^^21^ In a 3-year longitudinal study, increased CC flow voids (FVs) density has been shown to correlate with DR and DME progression.^22^

Prior studies have reported that reduced retina perfusion correlated with DR progression.^23^^,^^24^ Microvascular damage in DR results in decreased perfusion, leading to tissue ischemia and functional impairment. This perfusion abnormality contributes to breakdown of the blood retinal barrier, further accelerating DR progression.^25^ Recent studies focusing on changes in microvascular perfusion density (PD) in patients with DR have demonstrated a noninvasive approach to quantify microvascular alterations and enable early detection of DR-related microvascular changes.^26^^,^^27^

Beyond capillary-level alterations, changes in larger retinal vessels have also been implicated in DR pathophysiology. Our group developed an algorithm capable of detecting changes in both microvascular and large vessels (LVs), as well as quantifying variations in LV parameters, recognizing that these alterations differ across varying severities of DR.^28^ The association of dilation of larger retinal vessels with increasing DR severity has been reported and the changes in CC with DR severity have been previously reported by our group. Therefore, evaluation of perfusion on OCTA differentiating between retinal capillaries (CP), the larger arterioles and venules, and CC is important in differentiating DR severity.^29^ Collectively, these findings suggest that comprehensive evaluation of retinal perfusion using OCTA—differentiating between retinal CP, larger arterioles and venules, and CC—may provide complementary information for characterizing DR severity. Accordingly, in our study, we analyzed parameters from different retinal layers, focusing on both CP and larger vessels, as they exhibit distinct changes across varying DR severities.

Despite increasing interest in OCTA-derived vascular metrics, prospective longitudinal data linking baseline OCTA parameters to subsequent changes in DR status and visual outcomes remain limited. In this prospective cohort study, we aimed to evaluate the associations between baseline OCTA-derived vascular parameters and 2-year DR progression and VA decline. We further assessed whether incorporating OCTA metrics could improve predictive model performance beyond traditional systemic risk factors. We hypothesized that segmentation of different vascular components, including retinal CP, larger retinal vessels, and CC flow metrics, would enhance the ability to predict DR progression beyond systemic biomarkers or baseline DR severity alone.

## Methods

### Participants

We conducted a prospective cohort study from April 2018 to July 2019 at the Singapore National Eye Centre (SNEC) clinics. Written informed consent was obtained from all participants. Patients with DM were enrolled based on the following criteria: (1) age ≥21 years; (2) type 2 diabetes for >5 years; and (3) no end-stage renal failure. The exclusion criteria included glaucoma, age-related macular degeneration, significant media opacity, OCTA signal strength <7, and axial eye length >26.5 mm. This study followed the Strengthening the Reporting of Observational Studies in Epidemiology reporting guideline. Ethics approval for this study was obtained from the review board of Singapore Nation Eye Centre (Institutional Review Board No: 2017-2562). The study adhered to the tenets of the Declaration of Helsinki. Institutional Review Board/Ethics Committee approval was obtained.

All baseline examinations were conducted between April 2018 and July 2019. Participants were prospectively followed for 2 years, with follow-up visits performed at predefined intervals irrespective of baseline DR severity. For the present longitudinal analysis, 2 time points were included: baseline and the final follow-up visit at 2 years. Only eyes with complete baseline and 2-year follow-up data were included in the longitudinal analysis.

### OCTA

**Figure 1:**
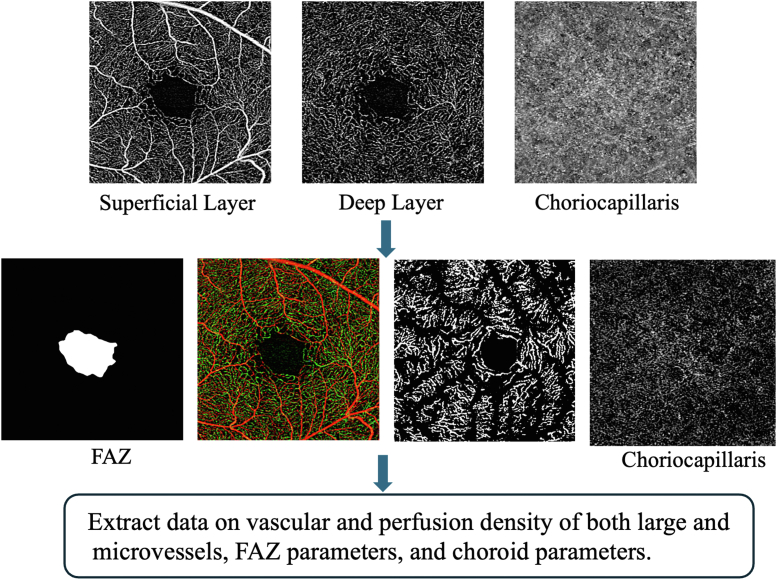
OCT angiography image processing and quantitative extraction workflow. FAZ = foveal avascular zone.

We utilized a prototype swept-source OCTA system (PlexElite 9000, Zeiss Meditec) with a wavelength scanning laser (λc = 1050 nm) as its light source. The system performs at a speed of 200kHx A-scans per second, with axial and lateral resolutions of 6.3 μm and 20 μm in tissue, respectively. All the OCTA scans in our study were conducted by experienced ophthalmic technicians at SNEC. We have described details of OCTA procedures in a previously published article.^30^ A 3 × 3 mm^2^ area centered at the fovea was scanned, and OCTA images were generated using a microangiography algorithm.^31^ PlexElite Review Software (Version 1.6, Zeiss Meditec) was utilized to segment the retinal pigment epithelium and retinal layers automatically. Incorrect auto segmentations were corrected and resegmented manually. Segmentations of the CC layer were conducted by using a standard protocol developed by Spaide (31–39 μm beneath the retinal pigment epithelium). Image signal weaker than 7, with poor quality and ungradable artefacts, will be excluded followed by standard criteria.^32^ Our detailed protocols have been published previously^33^ (Fig 1).

### OCTA Metrics

A MATLAB algorithm (The MathWorks, Inc) was utilized to extract quantitative OCTA metrics from the scan. To enhance the contrast of LVs, we applied a combined Gabor and Hessian–based vessel filter, followed by a thresholding method to generate a binarized mask.

The foveal avascular zone (FAZ) was manually delineated on en face OCTA images from both the superficial and deep vascular plexuses. Foveal avascular zone area was calculated from the enclosed region, and FAZ perimeter was measured as the total length of the FAZ boundary. Foveal avascular zone circularity was calculated using the standard formula 4π × FAZ area/(FAZ perimeter),^2^ with values closer to 1 indicating a more regular and circular FAZ.

The annulus was defined as a concentric ring region centered on the fovea, extending from the boundary of the FAZ to a predefined outer radius, and was used for quantitative analysis of vascular parameters. The following 4 vascular parameters were calculated: PD, vessel density (VD), large VD (LVD), and CC FVs density. Vessel density in each annulus was determined by the total vessel length divided by the total annulus area. Perfusion density and VD were analyzed as complementary metrics, as PD reflects the proportion of perfused vascular area, whereas VD represents total vessel length normalized to the analyzed area and is more sensitive to changes in capillary rarefaction and vascular remodeling. Choriocapillaris FV refers to areas with absent detectable blood flow in the CC layer. Perfusion density and VD were calculated for the entire vascular network, for LVs and for CP after exclusion of LVs. We calculated CC FV separately for various size thresholds (>200 μm^2^, >400 μm^2^, >600 μm^2^, and >800 μm^2^). These multiple size thresholds were used to capture FVs of different spatial scales, as smaller and larger FVs may reflect varying degrees of CC impairment and to reduce the influence of noise and segmentation artifacts. Using the binarized mask, we calculated PD and VD both with and without LVs. Choriocapillaris FV density was determined by calculating the percentage of the image area occupied by FVs, excluding regions containing LVs. In addition, CC FVs were identified using a standardized binarization threshold and analyzed across multiple size thresholds to minimize the influence of noise and segmentation-related artifacts. For PD and CP density, higher values indicated better vessel integrity, whereas higher FV values reflected poorer vascular distribution. Additionally, a higher VD value indicated greater vessel tortuosity. We defined the no LVs parameters as CP parameters. Choriocapillaris vessel information was extracted from morphological images, with binarization performed using a threshold of mean - standard deviation.^34^ For retinal vascular analysis, en face OCTA images were binarized to generate vessel masks for PD calculation. The binarized images were subsequently skeletonized to extract the 1-pixel-wide centerlines of the vessels, from which total vessel length was obtained. Vessel density was defined as the total vessel length divided by the analyzed area. The image processing workflow is illustrated in Supplementary Figure S1, available at www.ophthalmologyscience.org. The detailed protocol has been previously published by our team.^28^

Diabetic retinopathy progression was determined based on standardized grading of color fundus acquired at baseline and follow-up visits. All retinal images were obtained using standardized imaging protocols. Diabetic retinopathy severity was graded according to the ETDRS severity scale by trained graders who were masked to clinical data and OCTA findings from SNEC. Diabetic retinopathy progression was defined as a ≥2-step worsening on the ETDRS severity scale during follow-up.

### Statistics

We used means and standard deviations to describe the quantitative variables and numbers and percentages for the categorical variables. To account for correlations between eyes, we employed generalized estimating equation models to compare the differences in baseline characteristics and distribution of OCTA variables between the DR progression and nonprogression groups, VA worsening and non-VA worsening, adjusting for the correlation between eyes.

Multivariate logistic regression models were constructed to assess the relationship between baseline OCTA metrics and the risk of DR progression, by adjusting for established risk factors (age, diabetes duration, glycated hemoglobin [HbA1c], mean arterial pressure, and DR severity).^35^^,^^36^ Additionally, we defined VA decline by >1 line as the secondary outcome. From the baseline data, we conducted a simple linear regression model. Considering the correlations between eyes, we identified significant systemic factors, including diastolic blood pressure, body mass index (BMI), HbA1c, baseline best-corrected VA, serum glucose levels, and DR severity. These factors were incorporated into the model for adjustment to ensure robust predictions.

### VA Worsening and DR Progression Group

In our study, DR progression was defined as an increase of ≥2 steps in DR severity from baseline, based on the ETDRS grading scale.^37^ We categorized ETDRS levels into simplified steps as follows: level 10 as step 1, levels 15 and 20 as step 2, level 35 as step 3, level 43 as step 4, and level 47 as step 5. Examinations were performed by experienced technicians at the SNEC. Visual acuity decline was defined as a decrease in best-corrected VA of >1 line compared with the baseline.

OCT angiography variables for both the retina and choroid were added to the prediction models to evaluate whether these variables could enhance the models' predictive performance. Both established and proposed factors were included separately in the models, allowing us to assess the independent contributions of these OCTA metrics to DR progression and VA decrease. To evaluate the performance of the prediction models, we calculated the area under the receiver operating characteristic curve (AUC). A higher AUC value, closer to 1, indicates superior discriminative ability of the model in predicting DR progression. Brier score was utilized to assess the accuracy of the models. The score measures prediction errors by calculating the mean squared error between the observed and predicted outcome and actual outcomes ranging from 0 to 1. Lower Brier score means the model with better calibration and performance. A *P* value >0.05 indicates that the discrepancies between observed and expected outcomes are not statistically significant, suggesting that the model fits the data well.

## Results

### Baseline Characteristics

**Figure 2:**
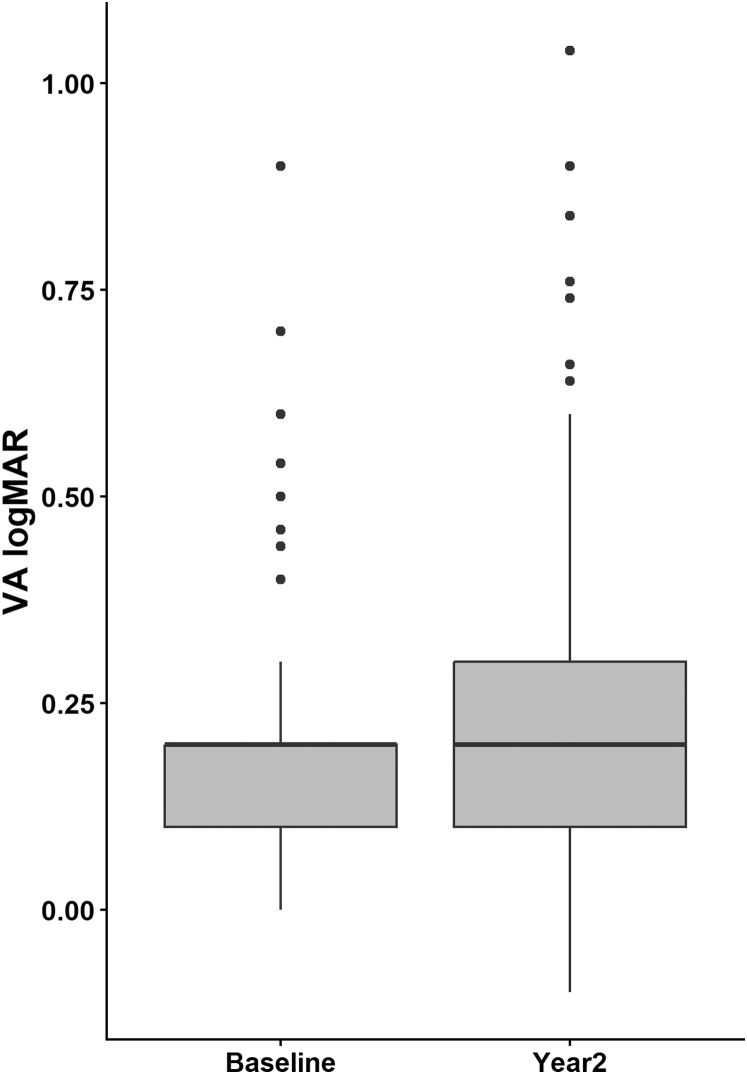
Illustrates the VA of patients with diabetic retinopathy progression at baseline and at year 2. logMAR = logarithm of the minimum angle of resolution; VA = visual acuity.

We included a total of 309 eyes from 192 individuals in our study. Over the course of 2 years, 260 eyes were in the no-progression group, and 49 eyes were in the progression group. Table 1 presents the demographic factors for the no-progression and progression groups. Patients in the progression group exhibited a higher BMI (27.00 vs. 25.76, *P* = 0.044), a higher baseline HbA1c level (8.49% vs. 7.90%, *P* = 0.031), and a shorter axial length (AL) (23.75 mm vs. 24.13 mm, *P* = 0.033). Other variables like age, gender, race, DM duration, mean arterial pressure, smoking status, hypertension did not show significant difference between groups. Supplementary Table S1A, available at www.ophthalmologyscience.org, provides details on the progression of DR across different stages. Supplementary Table S1B, available at www.ophthalmologyscience.org, illustrates the onset of new DME in patients with vision decline, with 1 patient who had no DME at baseline progressing to DME by year 2. Figure 2 shows VA of patients with DR progression at baseline and year 2.

**Table 1:** Characteristics for Participants by DR Progression and VA Worsening

Factors	No Progression N = 260	Progression N = 49	*P* Value	No VA Worsening N = 267	VA Worsening N = 40	*P* Value
Age (years), (SD)	62.71 (9.03)	60.10 (10.15)	0.096	62.14 (9.42)	62.90 (8.12)	0.69
Gender (female, %)	62.71 (9.03)	60.10 (10.15)	0.655	85 (31.8)	13 (32.5)	0.983
Race						
Chinese	216 (83.1)	40 (81.6)	0.792	223 (83.5)	31 (77.5)	0.789
Indian	24 (9.2)	5 (10.2)		26 (9.7)	3 (7.5)	
Malay	16 (6.2)	4 (8.2)		15 (5.6)	5 (12.5)	
Others	4 (1.5)			3 (1.1)	1 (2.5)	
DM duration (years)	17.41 (9.45)	17.04 (10.94)	0.75	17.33 (9.83)	17.32 (9.01)	0.96
Mean arterial pressure, mmHg	97.14 (12.28)	96.96 (12.12)	0.984	97.49 (12.33)	94.44 (11.70)	0.115
Body mass index	25.76 (4.05)	27.00 (4.73)	**0.044**	25.71 (4.00)	27.55 (5.10)	**0.009**
Smoking status, yes	80 (30.8)	13 (26.5)	0.42	79 (29.6)	14 (35.0)	0.515
Hypertension, yes	196 (75.4)	38 (77.6)	0.2	199 (74.5)	33 (82.5)	0.31
Diabetic nephropathy, yes	50 (19.2)	12 (24.5)	0.578	51 (19.1)	11 (27.5)	0.276
Glucose serum, mmol/L	10.19 (4.76)	10.37 (4.07)	0.906	10.21 (4.58)	10.30 (5.21)	0.806
HbA1c, %	7.90 (1.44)	8.49 (1.67)	**0.031**	7.98 (1.48)	8.16 (1.56)	0.433
Total cholesterol, mmol/L	4.36 (1.17)	4.31 (0.95)	0.084	4.40 (1.18)	4.15 (0.77)	0.151
HDL-c, mmol/L	1.25 (0.31)	1.23 (0.26)	0.692	1.26 (0.29)	1.20 (0.33)	0.318
Triglyceride, mmol/L	2.38 (1.77)	2.32 (1.35)	0.764	2.40 (1.80)	2.26 (1.01)	0.561
LDL-c, mmol/L	2.48 (0.80)	2.49 (0.82)	0.841	2.51 (0.81)	2.31 (0.76)	0.195
Cholesterol/HDL ratio	3.61 (0.99)	3.58 (0.82)	0.902	3.60 (0.96)	3.66 (1.02)	0.657
eGFR, mL/min/1.73 m^2^	78.90 (22.11)	79.34 (24.02)	0.89	80.02 (22.34)	72.71 (22.20)	**0.06**
Best-corrected visual acuity, logMAR	0.29 (0.20)	0.28 (0.21)	0.891	0.21 (0.14)	0.15 (0.13)	**0.048**
Intraocular pressure, mmHg	17.51 (3.19)	17.63 (2.98)	0.931	17.75 (3.13)	16.00 (3.03)	**0.003**
Sphere equivalent, D	–0.86 (2.07)	–0.44 (1.35)	0.152	–0.77 (1.89)	–0.82 (2.51)	0.83
Axial length, mm	24.13 (1.31)	23.75 (0.98)	**0.033**	24.04 (1.23)	24.12 (1.42)	0.673
DR severity						
No DR	99 (38.1)	19 (38.8)	0.461	99 (37.1)	17 (42.5)	0.532
Mild NPDR	101 (38.8)	16 (32.7)		104 (39.0)	13 (32.5)	
Moderate NPDR	60 (23.1)	14 (28.6)		64 (24.0)	10 (25.0)	

DM = diabetes mellitus; DR = diabetic retinopathy; eGFR = extimated glomerular filtration rate; HbA1c = glycated hemoglobin; HDL-c = high-density lipoprotein cholesterol; LDL-c = low-density lipoprotein cholesterol; logMAR = logarithm of the minimum angle of resolution; NPDR = nonproliferative diabetic retinopathy; SD = standard deviation; VA = visual acuity.

Bold *P* value: *P* < 0.05.

Table 2 demonstrates the OCTA metrics between the groups. At baseline, patients with DR progression had a longer FAZ perimeter (3.38 [1.40] vs. 2.80 [0.94], *P* = 0.002) and worse FAZ circularity (1.50 [0.39] vs. 1.37 [0.29], *P* = 0.011) in the superficial layer. Both LV (8.46 [1.49] vs. 7.77 [1.41], *P* = 0.004) and CP (17.66 [2.67] vs. 18.65 [2.68], *P* = 0.038) PD increased in the progression group. Vessel density also showed an increase in the progression group for LVs (4.03 [0.83] vs. 3.70 [0.55], *P* = 0.002) and a decrease for CP (14.21 [2.57] vs. 15.40 [2.66], *P* = 0.018). No significant differences were observed in FAZ perimeters, PD, or VD in the deep layer.

**Table 2:** OCTA Variables by DR Progression and VA Worsening

Variables	No Progression	Progression	*P* Value	No VA Worsening	Worsening VA	*P* Value
	N = 43	N = 149		N = 267	N = 40	
CC_FV_density	17.26 (1.49)	17.49 (1.56)	0.269	17.26 (1.50)	17.38 (1.40)	0.691
CC_FV_density_200	15.62 (1.41)	15.94 (1.44)	0.159	15.64 (1.39)	15.74 (1.45)	0.672
CC_FV_density_400	13.23 (1.59)	13.63 (1.57)	0.108	13.25 (1.54)	13.41 (1.77)	0.542
CC_FV_density_600	11.35 (1.76)	11.76 (1.71)	0.123	11.35 (1.69)	11.61 (2.00)	0.374
CC_FV_density_800	9.86 (1.85)	10.29 (1.82)	0.127	9.86 (1.77)	10.21 (2.15)	0.252
CC_FV_size	447.95 (85.26)	467.43 (96.87)	0.219	448.14 (80.85)	465.22 (121.70)	0.273
CC_FV_number	3342.41 (913.07)	3221.27 (845.87)	0.595	3332.70 (899.89)	3276.20 (948.11)	0.673
Superficial capillary plexus						
FAZ area	0.34 (0.17)	0.40 (0.18)	0.074	0.35 (0.15)	0.37 (0.27)	0.681
FAZ perimeter	2.80 (0.94)	3.38 (1.40)	**0.002**	2.90 (1.07)	2.86 (0.88)	0.490
FAZ circularity	1.37 (0.29)	1.50 (0.39)	**0.011**	1.39 (0.31)	1.38 (0.30)	0.632
Deep capillary plexus						
FAZ area	1.63 (0.67)	1.52 (0.50)	0.269	1.61 (0.65)	1.62 (0.57)	0.991
FAZ perimeter	7.23 (3.63)	6.65 (2.31)	0.162	7.15 (3.45)	7.18 (3.49)	0.974
FAZ circularity	1.65 (0.72)	1.59 (0.65)	0.521	1.64 (0.70)	1.64 (0.77)	0.981
SCP_LVD_PD	7.77 (1.41)	8.46 (1.49)	**0.004**	7.80 (1.44)	8.37 (1.42)	**0.041**
SCP_PD	26.33 (2.03)	26.02 (1.75)	0.357	26.38 (1.86)	25.99 (2.14)	0.281
SCP_CP_PD	18.65 (2.68)	17.66 (2.67)	**0.038**	18.67 (2.56)	17.75 (2.90)	**0.080**
SCP_LVD_VD	3.70 (0.55)	4.03 (0.83)	**0.002**	3.71 (0.61)	3.95 (0.62)	**0.031**
SCP_VD	19.22 (2.30)	18.37 (2.04)	**0.042**	19.17 (2.17)	18.83 (2.54)	0.401
SCP_CP_VD	15.40 (2.66)	14.21 (2.57)	**0.018**	15.34 (2.56)	14.76 (2.92)	0.261
DCP_PD	22.83 (3.15)	22.70 (3.10)	0.919	22.81 (3.12)	22.96 (3.15)	0.780
DCP_VD	16.55 (3.43)	16.17 (3.24)	0.600	16.47 (3.37)	16.74 (3.49)	0.760

CC = choriocapillaris; CP = capillaries; DCP = deep-layer capillary plexus; DR = diabetic retinopathy; FAZ = foveal avascular zone; FV = flow void; LVD = large vessel density; OCTA = OCT angiography; PD = perfusion density; SCP = superficial capillary plexus; VA = visual acuity; VD = vessel density.

Bold values indicate statistical significance (*P* < 0.05).

For the secondary outcome, in the superficial layer, the LV PD was significantly higher in the VA worsening group (8.37 [1.42] vs. 7.80 [1.44], *P* = 0.04), while the PD excluding LVs was smaller in the same group (17.75 [2.90] vs. 18.67 [2.56], *P* = 0.08). Additionally, the large-vessel VD was significantly higher in the VA worsening group (3.95 [0.62] vs. 3.71 [0.61], *P* = 0.03).

### Correlations between OCTA Parameters and DR Progression

Table 3 presents the results of the univariate and multivariate regression analyses examining the association between OCTA variables and both DR progression and VA decline. After adjusting for selected risk factors, a larger baseline FAZ area was significantly associated with DR progression (odds ratio [OR]: 6.612; 95% confidence interval [CI]: 1.152–37.964; *P* = 0.034). Additionally, longer FAZ perimeter (OR: 1.583; 95% CI: 1.188–2.110; *P* = 0.002) and worse FAZ circularity (OR: 3.23; 95% CI: 1.21–8.623; *P* = 0.019) were significantly associated with DR progression. No significant associations were found between CC flow deficit parameters and DR progression.

**Table 3:** Relationships between OCTA Variables and Risk of DR Progression and VA Worsening

OCTA Variables	DR Progression				VA Worsening			
	Univariate		Multivariate∗		Univariate		Multivariate†	
	OR (95% CI)	*P* Value	OR (95% CI)	*P* Value	OR (95% CI)	*P* Value	OR (95% CI)	*P* Value
CC_FV_density	1.1 (0.912–1.327)	0.320	1.114 (0.915–1.356)	0.284	1.053 (0.852–1.302)	0.633	1.054 (0.838–1.325)	0.654
CC_FV_density_200	1.156 (0.945–1.413)	0.158	1.147 (0.931–1.413)	0.199	1.05 (0.834–1.322)	0.677	1.039 (0.807–1.338)	0.765
CC_FV_density_400	1.159 (0.966–1.391)	0.113	1.144 (0.944–1.386)	0.169	1.064 (0.866–1.307)	0.553	1.067 (0.845–1.347)	0.588
CC_FV_density_600	1.139 (0.963–1.346)	0.128	1.132 (0.949–1.351)	0.168	1.089 (0.904–1.311)	0.369	1.113 (0.9–1.375)	0.323
CC_FV_density_800	1.13 (0.964–1.325)	0.133	1.13 (0.954–1.338)	0.157	1.108 (0.93–1.321)	0.251	1.142 (0.936–1.395)	0.192
CC_FV_size	1.002 (0.999–1.006)	0.153	1.002 (0.999–1.006)	0.241	1.002 (0.998–1.006)	0.248	1.003 (0.999–1.007)	0.188
CC_FV_number	1 (0.999–1)	0.393	1 (0.999–1)	0.342	1 (1–1)	0.713	1 (0.999–1)	0.783
Superficial capillary plexus								
FAZ area	5.662 (1.14–28.122)	**0.034**	6.612 (1.152–37.964)	**0.034**	1.928 (0.338–10.979)	0.460	4.186 (0.531–33.003)	0.174
FAZ perimeter	1.539 (1.195–1.984)	**<0.001**	1.583 (1.188–2.110)	**0.002**	0.957 (0.689–1.329)	0.793	1.022 (0.67–1.559)	0.919
FAZ circularity	3.209 (1.317–7.822)	**0.01**	3.23 (1.21–8.623)	**0.019**	0.888 (0.296–2.661)	0.831	0.847 (0.227–3.159)	0.805
SCP_LV_PD	1.411 (1.129–1.763)	**0.003**	1.561 (1.212–2.01)	**<0.001**	1.321 (1.042–1.673)	**0.021**	1.609 (1.198–2.162)	**0.002**
SCP_PD	0.924 (0.795–1.074)	0.305	0.88 (0.743–1.042)	0.14	0.897 (0.755–1.067)	0.219	0.791 (0.635–0.985)	**0.036**
SCP_CP_PD	0.871 (0.776–0.977)	**0.019**	0.825 (0.725–0.94)	**0.004**	0.872 (0.765–0.994)	**0.040**	0.8 (0.676–0.947)	**0.010**
SCP_LV_VD	2.359 (1.422–3.911)	**<0.001**	2.878 (1.622–5.107)	**<0.001**	1.793 (1.066–3.016)	**0.028**	2.172 (1.203–3.921)	**0.010**
SCP_VD	0.831 (0.715–0.967)	**0.016**	0.798 (0.673–0.947)	**0.010**	0.937 (0.813–1.081)	0.373	0.864 (0.725–1.031)	0.10
SCP_CP_VD	0.826 (0.725–0.942)	**0.004**	0.792 (0.684–0.917)	**0.002**	0.912 (0.785–1.061)	0.233	0.841 (0.696–1.016)	0.071
Deep capillary plexus								
FAZ area	0.765 (0.461–1.267)	0.298	0.799 (0.463–1.379)	0.421	1.009 (0.601–1.694)	0.974	1.16 (0.586–2.293)	0.671
FAZ perimeter	0.945 (0.854–1.047)	0.279	0.951 (0.853–1.06)	0.365	1.003 (0.911–1.104)	0.954	1.01 (0.873–1.169)	0.891
FAZ circularity	0.885 (0.566–1.384)	0.592	0.903 (0.559–1.458)	0.676	1.008 (0.63–1.614)	0.972	1.027 (0.571–1.847)	0.93
DCP_PD	0.987 (0.895–1.088)	0.793	0.986 (0.887–1.096)	0.798	1.016 (0.914–1.131)	0.764	0.998 (0.889–1.12)	0.970
DCP_VD	0.967 (0.881–1.06)	0.471	0.968 (0.877–1.07)	0.528	1.023 (0.929–1.127)	0.643	1.022 (0.919–1.136)	0.692

BMI = body mass index; CC = choriocapillaris; CI = confidence interval; CP = capillaries; DCP = deep-layer capillary plexus; DR = diabetic retinopathy; FAZ = foveal avascular zone; FV = flow void; HbA1c = glycated hemoglobin; HDL = high-density lipoprotein; LV = large vessel; OCTA = OCT angiography; OR = odds ratio; PD = perfusion density; SCP = superficial capillary plexus; VA = visual acuity; VD = vessel density.

Bold values indicate statistical significance (*P* < 0.05).

∗ Multivariate model: adjusted for age, diabetes duration, pulse rate, mean arterial pressure, HbA1c, cholesterol/HDL ratio, and baseline DR severity.

† Multivariate model: variables were first selected using mixed-effects models accounting for inter-eye correlation. The final model was adjusted for for diastolic blood pressure, BMI, HbA1c, baseline best-corrected VA, and DR severity.

In the superficial layer, higher LV PD (OR: 1.561; 95% CI: 1.212–2.01; *P* < 0.001) was significantly associated with DR progression, while lower CP PD (OR: 0.825; 95% CI: 0.725–0.94; *P* = 0.004) also showed a significant association. Higher VD for LVs (OR: 2.878; 95% CI: 1.622–5.107; *P* < 0.001), as well as for the whole-vessel network (OR: 0.798; 95% CI: 0.673–0.947; *P* = 0.010) and for CP (OR: 0.792; 95% CI: 0.684–0.917; *P* = 0.002), were associated with DR progression. No significant association was observed between PD or VD in the deep capillary plexus (DCP) and DR progression.

After adjusting for covariates that showed significant differences at baseline, in the superficial layer, higher LV PD was associated with VA decline (OR: 1.609; 95% CI: 1.198–2.162; *P* = 0.002). In contrast, higher capillary PD was associated with no VA decline (OR: 0.800; 95% CI: 0.676–0.947; *P* = 0.010). Similarly, the total superficial layer PD also showed a negative correlation with VA decline (OR: 0.791; 95% CI: 0.635–0.985; *P* = 0.036).

### Performance of the Model for Prediction of DR Progression and VA Decline

**Figure 3:**
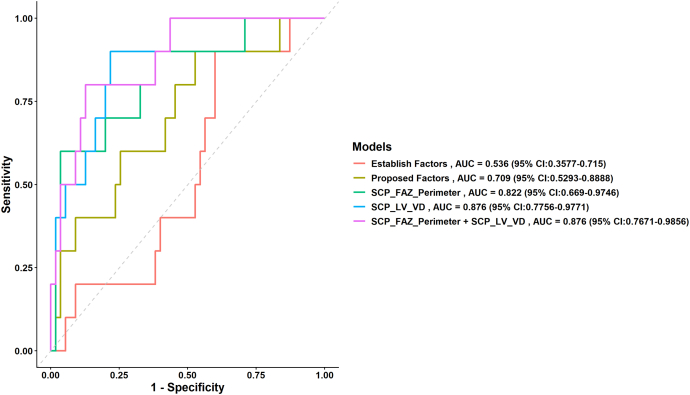
Receiver operating characteristic curve of prediction models for incidence of DR progression. The establish factors were age, DM duration, HbA1c, MAP, and DR severity. Proposed factors were AL + BMI + HbA1c + DR severity. The SCP_FAZ_perimeter model was proposed factors combined with SCP_FAZ_perimeter. The SCP_LV_VD model was proposed factors combined with SCP_LV_VD. The SCP_FAZ_perimeter + SCP_LV_VD model was proposed factors. AL = axial length; AUC = area under the receiver operating characteristic curve; BMI = body mass index; CI = confidence interval; DM = diabetes mellitus; DR = diabetic retinopathy; FAZ = foveal avascular zone; HbA1c = glycated hemoglobin; LV = large vessel; MAP = mean arterial pressure; MAP = mean arterial pressure; SCP = superficial capillary plexus; VD = vessel density.

Table 4 presents the predictive performance of our model after adjusting for the proposed risk factors selected from our dataset and incorporating OCTA metrics. Model 1 includes established risk factors from previously published studies: age, DM duration, HbA1c, mean arterial pressure, and DR severity. This model achieved an AUC of 0.536, a Brier score of 0.135, and a Hosmer–Lemeshow (HL) *P* value of 0.039. Model 2 includes the proposed risk factors identified in Table 1 that are associated with DR progression: AL, BMI, HbA1c, and DR severity. This model demonstrated an improved predictive performance, with an AUC of 0.709, an HL *P* value of 0.587, and a Brier score of 0.124. Regression models incorporating additional OCTA variables, specifically superficial capillary plexus (SCP)_FAZ_perimeter (model 3) and SCP_LV_VD (model 4), demonstrated improved predictive performance, with higher AUC values of 0.822 and 0.876, respectively, and lower Brier scores of 0.108 and 0.104. When SCP_FAZ_perimeter and SCP_LV_VD were combined with the proposed risk factors (model 5), the model achieved an AUC of 0.876 and an even better calibration with a Brier score of 0.100. Figure 3 demonstrates the performance of the models employed in our study.

**Table 4:** Prediction Performance of Models for DR Progression

Model	Multivariate OR (CI)	AUC	HL	Brier Score	*P* Value
Model 1		0.536	0.039	0.135	
Model 2		0.709	0.587	0.124	
Model 3	1.583 (1.188–2.11)∗	0.822	0.489	0.108	0.051
Model 4	2.878 (1.622–5.107)∗	0.876	0.691	0.104	0.057
Model 5		0.876	0.6	0.100	0.036

AL = axial length; AUC = area under the receiver operating characteristic curve; BMI = body mass index; CI = confidence interval; DM = diabetes mellitus; DR = diabetic retinopathy; FAZ = foveal avascular zone; HbA1c = glycated hemoglobin; HL = Hosmer–Lemeshow; LV = large vessel; MAP = mean arterial pressure; OR = odds ratio; VD = vessel density.

Model 1: adjusted for established factors: age + DM duration + HbA1c + MAP + DR severity.

Model 2: adjusted for our proposed factors: AL + BMI + HbA1c + DR severity.

Model 3: model 2 + SCP_FAZ_perimeter.

Model 4: model 2 + SCP_LV_VD.

Model 5: model 2 + SCP_FAZ_Perimeter + SCP_LV_VD.

AUC: measures the ability of the model to distinguish between classes. Higher values indicate better performance.

Hosmer–Lemeshow test: Assesses the goodness of fit for logistic regression models; *P* > 0.05 indicates good fit.

Brier score: measures the accuracy of probabilistic predictions; lower values indicate better performance.

∗ *P* < 0.05.

**Figure 4:**
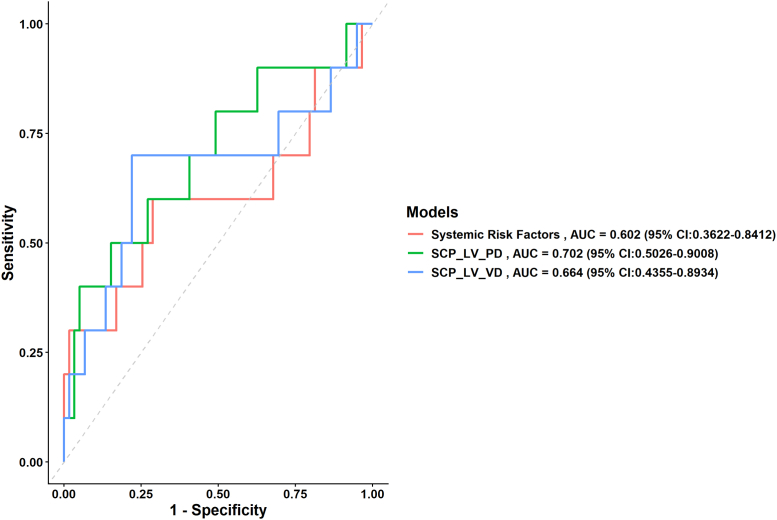
Receiver operating characteristic curve of prediction models for incidence of VA decline. The systemic risk factors were DBP + BMI + HbA1c + baseline best VA + DR severity. The SCP_LV_PD model was systemic factors combined with SCP_LV_PD. The SCP_LV_VD model was systemic factors combined with SCP_LV_VD. AUC = area under the receiver operating characteristic curve; BMI = body mass index; CI = confidence interval; DBP = diastolic blood pressure; DR = diabetic retinopathy; HbA1c = glycated hemoglobin; LV = large vessel; PD = perfusion density; SCP = superficial capillary plexus; VA = visual acuity; VD = vessel density.

Supplementary Table S2, available at www.ophthalmologyscience.org, showed prediction performance of models using VA decline outcome. Model 1 achieved an AUC of 0.602, indicating moderate discriminative ability, with an HL statistic of 0.462 and a Brier score of 0.116, suggesting reasonable calibration and prediction accuracy. Model 2, which incorporated additional predictors as LV PD in superficial layer, showed improved performance with an OR of 1.6091 (95% CI: 1.198–2.162) and an increased AUC of 0.702. Model 3 further refined the prediction, achieving an OR of 2.172 (95% CI: 1.203–3.921), a slightly lower AUC of 0.664, and an HL statistic of 0.912, demonstrating strong calibration. The Brier score for model 3 was 0.113, slightly higher than that of model 2. Overall, both models 2 and 3 performed better than the baseline model, with model 2 offering the best combination of discrimination and calibration. Figure 4 illustrates the models we employed to predict the VA decline in our study. Stratified analyses by baseline lens status demonstrated that consistent associations between OCTA parameters and VA decline in both phakic and pseudophakic eyes (Supplementary Table S4, available at www.ophthalmologyscience.org).

## Discussion

Detecting DR progression before it progresses to proliferative DR is critical, as this progression can lead to visual impairment. Early identification of DR progression allows for timely intervention, potentially preventing severe vision loss in working age patients.^36^ In our 2-year prospective study, we found that OCTA parameters, including larger FAZ area, longer FAZ perimeter, worse circularity, and reduced PD in the retinal superficial layer, were significantly correlated with DR progression. Additionally, we found that the FAZ perimeter and LVD provided enhanced predictive performance for 2-year DR progression.

Body mass index and HbA1c have been consistently reported as risk factors for DR progression in prior studies.^17^^,^^38^ Supplementary Table S3, available at www.ophthalmologyscience.org, summarizes studies that incorporated various systemic factors into prediction models. These factors were associated with DM control and retinopathy progression. A 6-year observational study demonstrated that higher BMI is positively correlated with DR progression compared with patients with lower BMI.^39^ The association between DR progression and obesity remains unclear; we hypothesize that this may be influenced by ethnic differences and lifestyle factors. Given that early control of HbA1c has a “metabolic memory” effect on diabetic complications, DR can still progress even when short term glycemic levels are within the normal range.^40^ Optimizing long-term HbA1c control is crucial for preventing DR progression and should be a key focus for health systems.

Shorter AL was associated with DR progression in our cohort. Previous studies have reported a protective association between longer AL and DR incidence. For example, Ryan et al^41^ demonstrated that longer AL was associated with a lower risk of DR onset, with an OR of 0.58 per 1-mm increase in AL. A large cross-sectional study from China similarly reported a 19% decrease in DR prevalence for every 1-mm increase in AL.^42^ Although the underlying mechanisms remain incompletely understood, proposed explanations include dilution of intraocular VEGF due to increased vitreous volume and a higher likelihood of posterior vitreous detachment in eyes with longer AL.^43^ In addition, longer AL has been associated with a higher likelihood of posterior vitreous detachment, which has been proposed as a potential protective mechanism against DR. Given that AL was not a primary focus of the present study, these findings are discussed here for contextual interpretation. As AL may influence OCTA-derived perfusion and VD measurements through image magnification effects, AL was included as a covariate in the multivariable analyses, and the OCTA findings should be interpreted in this context.

The fovea with its central avascular zone has an intimate relationship with vison across various disease conditions.^44^ An increase in FAZ area has been positively correlated with DR progression, as demonstrated in both OCTA and fluorescein angiography studies.^45^^,^^46^ Enlargement or disruption of the FAZ and parafoveal capillary dropout are indicative of diabetic macular ischemia.^47^ Diabetic macular ischemia can be detected noninvasively by using OCTA, with the SCP area being associated with DR progression. In a 2-year follow-up study, researchers found that in their dataset, FAZ parameters in the DCP were associated with DR progression, while the SCP showed no correlation.^35^ We hypothesize that these discrepancies may partly reflect limitations in DCP measurement, including differences in OCTA metric algorithms and less distinct FAZ boundaries in the deep layer, which may reduce measurement reliability and reproducibility. Importantly, the FAZ area in the DCP is relatively large even in healthy eyes, such that subtle disease-related changes within an already large FV may be difficult to detect using FAZ-based DCP metrics alone. Consistent with this interpretation, Park et al^48^ found that the FAZ was most clearly delineated in the middle layer, while the deep layer was less well-defined compared with the middle layer. Histological studies have further demonstrated that the FAZ is surrounded by a specialized terminal capillary ring predominantly formed by CP from the superficial and intermediate plexuses, providing an anatomical basis for the stronger associations observed with SCP-related metrics in the present study.^30^^,^^49^ Although anatomical evidence suggests that the DCP may be preferentially affected in early diabetic microvascular damage, evaluating DCP perfusion outside the FAZ region may offer greater sensitivity. However, accurate assessment of DCP flow remains technically challenging due to projection artifacts from the superficial and intermediate plexuses.^50^

Despite accumulating evidence highlighting the role of the DCP in the pathogenesis and progression of DR, we did not observe significant associations between DCP-derived perfusion or VD metrics and DR progression in the present study. This null finding may reflect several factors. First, DCP measurements are more susceptible to projection artifacts and segmentation variability, which may reduce measurement sensitivity and reproducibility. Second, FAZ-based metrics in the DCP may be less sensitive to subtle disease-related changes, as the FAZ in the deep layer is relatively large even in healthy eyes. Finally, it is possible that early DCP alterations occur outside the central macular region captured by 3 × 3 mm OCTA scans, limiting our ability to detect longitudinal changes using the current imaging field. Future studies incorporating wider-field OCTA and improved artifact-correction algorithms may help better elucidate the role of DCP alterations in DR progression.

In our prediction model, we found that the FAZ perimeter can enhance the model’s predictive performance. Research suggests that the FAZ perimeter, which captures irregularities and shape changes, may be more sensitive to microvascular alterations. In contrast, the changes in FAZ area are less sensitive or may only occur in later stages of DR, and therefore have a weaker association with disease progression.^51^ Considering all FAZ metrics, rather than the area alone, may enhance the discriminative ability of the model which can better enable clinicians to identify those at risk of progression compared to DR status alone.

We found that adding LVD in the superficial layer could enhance the model's predictive performance. From a cross-sectional study, our colleague found that LVD differed across DR stages in OCTA scans, with higher density observed in the moderate to severe group compared with the mild group.^30^ Another group found that combining LV and microvascular characteristics significantly enhanced the model's classification performance.^52^ We have traditionally focused more on microvascular changes, such as microaneurysms, hemorrhages and cotton-wool spots, because DR is primarily considered a microangiopathy,^53^ and the ETDRS study found that capillary changes on fundus fluorescein angiography were weakly correlated with progression to proliferative DR.^54^ Our findings suggest that an increase density of LVs may precede and predict the capillary changes that characterize DR and could improve accuracy of predicting disease progression. This may be a similar effect to the prior literature suggesting an increase in retinal arteriolar caliber may predict incident DR. A plausible explanation is that dilation and remodeling of larger retinal vessels may reflect early hemodynamic dysregulation and venous congestion in diabetes, which may precede subsequent capillary dropout and microvascular damage.^55^ It should be noted that LVD was analyzed as a composite parameter without differentiation between arteries and veins. Given that venous dilation has been implicated in DR pathophysiology, future studies incorporating artery–vein differentiation using combined OCTA and fundus photography may further enhance the sensitivity and specificity of LV-based biomarkers.

Although several studies have shown that incorporating CC data can improve model performance, this characteristic did not improve the prediction of DR progression in our study.^21^^,^^22^^,^^30^ We believe this discrepancy may be attributed to differences in the algorithms used for extracting CC information, the varying characteristics of the participants, and the differences in variables adjusted for in the model.

Using VA decline as a secondary outcome, we found that prediction models performed poorer compared to DR progression. We chose a >1-line decline to increase sensitivity for detecting early functional changes over a 2-year follow-up period; however, alternative thresholds such as a >2-line decline may reduce measurement variability and should be explored in future studies. This may be attributed to several factors. Firstly, the VA can be influenced by multiple ocular and systemic variables beyond the DR status, such as cataract progression, refractive errors, and other retinal pathologies. These factors may potentially reduce the predictive accuracy of the models focused primarily on DR-specific parameters. Secondly, DR progression has often been observed by characters such as development of hemorrhages or proliferation, which can be linked with OCTA parameters such as vessel or PD and choroidal parameters. In contrast, VA decline represents the functional outcome, making it challenging to predict by using only structural biomarkers alone. Moreover, VA decline may vary between individuals, depending on factors such as compliance with treatment, DM control, and living environment. Despite these challenges, our findings underscore the potential value of integrating additional functional biomarkers, such as contrast sensitivity or low luminance VA, into prediction models to enhance the accuracy of VA outcomes. More studies with larger cohorts and longitudinal data may help refine prediction models for VA decline by incorporating a broader range of systemic and ocular parameters.^56^

Our model may have important implications for clinical practice and the health care system. Firstly, the model is adjusted for several significant factors identified in our dataset. These factors are easily accessible during routine clinical visits or retinal screenings. Additionally, OCTA parameters can be extracted noninvasively. In some less developed areas or for patients cannot be referred on time, this approach could help identify DR patients at higher risk of progression, allowing for targeted monitoring to prevent vision deterioration. Because FAZ perimeter is a reproducible OCTA metric, it is easily accessible to all clinicians, facilitating the detection or prediction of DR progression.

There are several limitations to our study. First, the follow-up period is relatively short for diabetic patients. A longer follow-up duration is necessary for future studies to better assess the long-term outcomes. Second, our study focused only on 3 × 3 mm OCTA scans, which limits our ability to assess peripheral vessel perfusion. A study published by our colleague demonstrated peripheral capillary dropout in nonproliferative DR using a 12 x 12 wide-field OCTA scan.^30^ The limited focus of the 3 x 3 field may have missed important information in the peripheral retinal vasculature. In addition, given the number of OCTA parameters evaluated, there is a potential risk of type I error due to multiple comparisons. Formal adjustment for multiple testing was not applied, and therefore the findings should be interpreted with appropriate caution.

Visual acuity decline may be influenced by factors beyond retinal microvascular changes, particularly lens status in an older DR population. Given that the mean age of our cohort was >60 years, cataract progression may have contributed to changes in VA during follow-up. To address this, we examined the impact of baseline lens status by performing stratified analyses in phakic and pseudophakic eyes, which demonstrated consistent directions of association between OCTA parameters and VA decline across both groups.

To further address the potential confounding effect of lens status on VA outcomes, we performed stratified analyses by baseline lens status. The direction and magnitude of associations between superficial LV-related OCTA metrics and VA decline were broadly consistent in both phakic and pseudophakic eyes, with stronger associations observed in pseudophakic eyes. This suggests that the observed relationships are unlikely to be driven solely by lens opacity and supports the robustness of LV OCTA parameters as imaging biomarkers for VA decline.

Although eyes with significant media opacity that precluded high-quality OCTA imaging were excluded at baseline, lens status was not systematically graded at each follow-up visit and cataract progression was not explicitly adjusted for in the analysis. This limitation is especially relevant for patients who required intravitreal injections, in whom cataract progression may be accelerated. Therefore, the associations between OCTA metrics and VA decline should be interpreted with consideration of this potential confounding factor.

## Conclusions

In conclusion, a larger FAZ area and longer FAZ perimeter in the superficial layer, higher LV perfusion, and VD, as well as lower capillary vessel perfusion and VD, were associated with a higher risk of DR progression over a 2-year period. Incorporating OCTA metrics and adjusting for our proposed factors—AL, BMI, HbA1c, DR severity, the superficial FAZ perimeter, and LVD—can enhance our model's predictive performance. For vision decline, increased LVD and PD in the superficial layer were associated with a higher risk of vision loss. OCT angiography parameters have demonstrated better predictive performance for DR progression compared to VA decline.
