# Supplementary material for: Using OCT Angiography to Predict Diabetic Retinopathy Progression and Vision Decline in a Multiethnic Cohort
Source: Ophthalmol Sci. 2026 Feb 24;6(5):101111. doi: 10.1016/j.xops.2026.101111 (PMC13059305; doi:10.1016/j.xops.2026.101111)
Supplement: Supplementary Table S1 [file mmc1.pdf]

Supplementary Table 4. Multivariable associations between baseline OCTA parameters and VA decline stratified by baseline lens status

OCTA parameter	Phakic eyes OR (95% CI)	P value	Pseudophakic eyes OR (95% CI)	P value
CC_FV_density	0.962 (0.751-1.231)	0.757	1.483 (0.909-2.42)	0.115
CC_FV_density_200	1.001 (0.779-1.286)	0.993	1.321 (0.694-2.516)	0.397
CC_FV_density_400	1.035 (0.816-1.312)	0.777	1.171 (0.686-1.998)	0.564
CC_FV_density_600	1.059 (0.848-1.323)	0.612	1.246 (0.787-1.974)	0.348
CC_FV_density_800	1.081 (0.872-1.34)	0.478	1.295 (0.852-1.97)	0.227
CC_FV_size	1.003 (0.998-1.008)	0.198	1.002 (0.994-1.009)	0.708
CC_FV_number	0.999 (0.999-1)	0.176	1 (1-1.001)	0.353
SCP_FAZ_area	0.406 (0.013-12.445)	0.606	66.296 (0.259-16957.379)	0.138
SCP_FAZ_perimeter	0.809 (0.47-1.39)	0.442	1.603 (0.807-3.183)	0.178
SCP_FAZ_circularity	0.594 (0.118-3.003)	0.529	0.883 (0.079-9.898)	0.92
DCP_FAZ_area	1.488 (0.744-2.976)	0.262	0.368 (0.094-1.443)	0.152
DCP_FAZ_perimeter	1.003 (0.893-1.126)	0.965	0.9 (0.67-1.208)	0.482
DCP_FAZ_circularity	0.93 (0.52-1.663)	0.808	0.994 (0.299-3.306)	0.992
SCP_LV_PD	1.484 (1.035-2.128)	**0.032**	2.004 (1.116-3.597)	**0.02**
SCP_LV_VD	1.649 (0.709-3.84)	0.246	4.483 (1.537-13.075)	**0.006**
DCP_ PD	0.959 (0.835-1.1)	0.546	1.074 (0.831-1.386)	0.587
DCP_ VD	0.966 (0.85-1.098)	0.596	1.131 (0.897-1.426)	0.298

Supplementary Table 4 presents the associations between baseline OCTA parameters and visual acuity decline (>0.1 logMAR) over two years, stratified by baseline lens status (phakic versus pseudophakic eyes). Odds ratios (ORs) and 95% confidence intervals (CIs) were derived from multivariable logistic regression models adjusted for diastolic blood pressure, body mass index, HbA1c, baseline best-corrected visual acuity (logMAR), and DR severity.
