# Supplementary material for: Using OCT Angiography to Predict Diabetic Retinopathy Progression and Vision Decline in a Multiethnic Cohort
Source: Ophthalmol Sci. 2026 Feb 24;6(5):101111. doi: 10.1016/j.xops.2026.101111 (PMC13059305; doi:10.1016/j.xops.2026.101111)
Supplement: Supplementary Table S2 [file mmc2.pdf]

Supplemantary **Table 2.** Prediction Performance of Models using VA outcome

Model	Multivariate OR (CI)	AUC	HL	Brier Score	P value
Model 1		0.602	0.462	0.116	
Model 2	1.609 (1.198-2.162)	0.702	0.629	0.11	0.263
Model 3	2.172 (1.203-3.921)	0.664	0.869	0.113	0.316

Supplementary Table 2. Prediction performance of multivariable logistic regression models for visual acuity (VA) decline over 2 years. Visual acuity decline was defined as a reduction of more than one line in best-corrected visual acuity. Model performance was evaluated using the area under the receiver operating characteristic curve (AUC), Hosmer–Lemeshow (HL) goodness-of-fit test, and Brier score.

Model 1 was adjusted for diastolic blood pressure (DBP), body mass index (BMI), glycated hemoglobin (HbA1c), baseline best logMAR visual acuity, and diabetic retinopathy (DR) severity.

Model 2 additionally included superficial capillary plexus large-vessel perfusion density (SCP LV PD).

Model 3 additionally included superficial capillary plexus large-vessel vessel density (SCP LV VD).

Odds ratios (ORs) are presented with 95% confidence intervals (CIs). Lower Brier scores indicate better model calibration.
