# Supplementary material for: Using OCT Angiography to Predict Diabetic Retinopathy Progression and Vision Decline in a Multiethnic Cohort
Source: Ophthalmol Sci. 2026 Feb 24;6(5):101111. doi: 10.1016/j.xops.2026.101111 (PMC13059305; doi:10.1016/j.xops.2026.101111)
Supplement: Supplementary Table S3 [file mmc3.pdf]

Supplementary **Table 1a** DR progression status in groups

**Baseline DR Severity**		**Year 2**									
		**10**	**15**	**20**	**35**	**43**	**47**	**53**	**65**	**71**	**90**
**No DR**	**10**	78	8	13	14	1	0	0	0	0	4
	**15**	4	1	0	3	0	0	0	0	0	3
Mild NPDR	**20**	10	3	3	19	4	1	0	0	0	1
	**35**	5	3	3	32	15	4	0	0	1	2
**Moderate NPDR**	**43**	2	0	1	15	17	10	3	0	1	7
	**47**	0	0	1	3	7	4	0	1	0	2

Supplementary Table 1b New DME (CRT>=320um) among the 40 VA worse patients

	**No DME yr2**	**DME yr2**	**Missing yr2**
**No DME baseline**	33	1	0
**DME baseline**	1	4	1

CRT: central retina thickness

Supplementary Table 1a. Progression of DR severity from baseline to year 2. Rows represent baseline DR severity categories and columns indicate DR severity at the 2-year follow-up based on the ETDRS classification. Values represent the number of eyes in each category.

Supplementary Table 1b. Incidence of new-onset DME among eyes with VA decline during follow-up. Values represent the number of eyes stratified by baseline and year-2 DME status.
