# Supplementary material for: Using OCT Angiography to Predict Diabetic Retinopathy Progression and Vision Decline in a Multiethnic Cohort
Source: Ophthalmol Sci. 2026 Feb 24;6(5):101111. doi: 10.1016/j.xops.2026.101111 (PMC13059305; doi:10.1016/j.xops.2026.101111)

(A) Original en face OCTA image

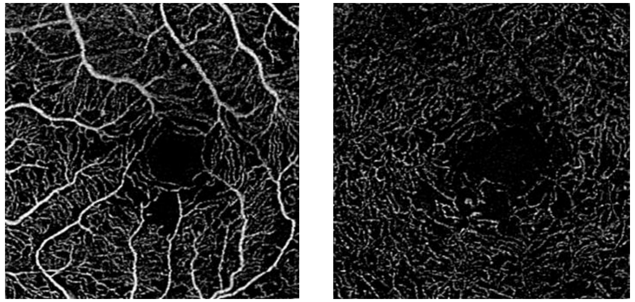

(B) Binarized vessel mask (for perfusion density)

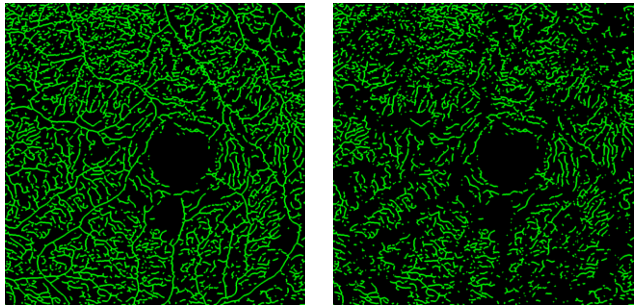

(C) Skeletonized vessels (for vessel density)

Vessel density (VD) = total vessel length / analyzed area

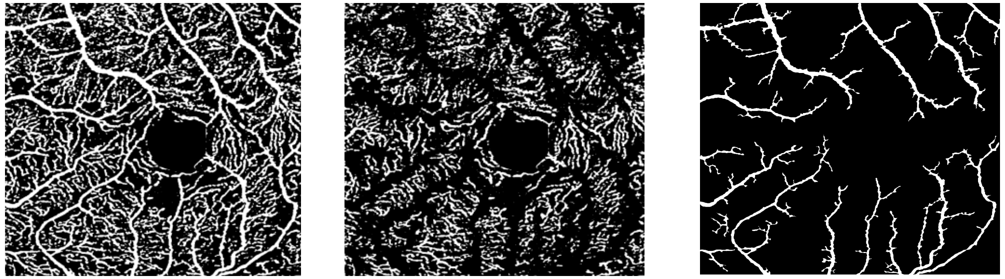

Supplement: Supplementary Table S4 [file mmc4.pdf]
